# MKI-1, a Novel Small-Molecule Inhibitor of MASTL, Exerts Antitumor and Radiosensitizer Activities Through PP2A Activation in Breast Cancer

**DOI:** 10.3389/fonc.2020.571601

**Published:** 2020-09-29

**Authors:** Ah-Young Kim, Yi Na Yoon, Jiyeon Leem, Jee-Young Lee, Kwan-Young Jung, Minsung Kang, Jiyeon Ahn, Sang-Gu Hwang, Jeong Su Oh, Jae-Sung Kim

**Affiliations:** ^1^Division of Radiation Biomedical Research, Korea Institute of Radiological and Medical Sciences, Seoul, South Korea; ^2^Radiological and Medico-Oncological Sciences, University of Science and Technology, Daejeon, South Korea; ^3^Department of Integrative Biotechnology, Sungkyunkwan University, Suwon, South Korea; ^4^New Drug Development Center, Daegu-Gyeongbuk Medical Innovation Foundation, Daegu, South Korea; ^5^Center for Medicinal Chemistry, Korea Research Institute of Chemical Technology, Daejeon, South Korea

**Keywords:** MASTL, PP2A, antitumor, breast cancer, radiosensitizer

## Abstract

Although MASTL (microtubule-associated serine/threonine kinase-like) is an attractive target for anticancer treatment, MASTL inhibitors with antitumor activity have not yet been reported. In this study, we have presented a novel MASTL inhibitor, MKI-1, identified through *in silico* screening and *in vitro* analysis. Our data revealed that MKI-1 exerted antitumor and radiosensitizer activities in *in vitro* and *in vivo* models of breast cancer. The mechanism of action of MKI-1 occurred through an increase in PP2A activity, which subsequently decreased the c-Myc protein content in breast cancer cells. Moreover, the activity of MKI-1 in the regulation of MASTL-PP2A was validated in a mouse oocyte model. Our results have demonstrated a new small-molecule inhibitor of MASTL, MKI-1, which exerts antitumor and radiosensitizer activities through PP2A activation in breast cancer *in vitro* and *in vivo.*

## Introduction

Mitosis is considered an attractive target for selective anticancer treatment owing to the dysregulation of mitotic kinases and checkpoints in cancerous cells compared with those in normal cells (1, 2). Many mitotic kinases, including CDKs, AURKs, and PLK1, are associated with tumor progression and prognosis in many types of cancers (1, 2); thus, small molecule inhibitors for mitotic kinases have been developed as anticancer drugs (1, 2). However, inhibitors against these mitotic kinases have produced disappointing clinical results, with poor therapeutic effects due to their cytotoxicity to healthy cells (1). Mitotic checkpoints have emerged as targets for selective anticancer treatment, as defects in mitotic checkpoints cause severe genomic instability and aneuploidy, selectively inducing the mitotic catastrophe in cancer cells (1, 3). Thus, factors regulating both mitosis and mitotic checkpoints, which are overexpressed in cancer cells, are promising targets for selective anticancer treatment.

Microtubule-associated serine/threonine kinase-like (MASTL), also known as Greatwall kinase, is a mitotic kinase involved in mitotic progression (1, 4). MASTL regulates mitosis and meiosis via the inactivation of the protein phosphatase 2A complex (PP2A-B55), through the direct phosphorylation of α-endosulfine (ENSA) and/or cAMP-regulated phosphoprotein 19; this is an essential mechanism to maintain high cyclin-B1-CDK1 activity during mitosis and meiosis in various model systems, including mammalian somatic cells and oocytes (5–9). Recent studies indicated that MASTL is overexpressed in several types of cancers, including breast, head and neck, thyroid, and colorectal cancers, and is associated with a poor prognosis, particularly in breast cancer (10–15). MASTL inhibition also reduced tumor growth and metastasis *in vitro* and *in vivo* in breast cancer (11–13). In addition, MASTL was reported to regulate several oncogenic properties, such as cellular transformation, chromosome instability, and metastasis (11, 13), and to regulate the DNA damage response and tumor resistance in response to anticancer treatments, such as radiotherapy, through the regulation of Chk2 (16–20). Moreover, MASTL inhibition caused mitotic cell death in various cancer cells, with less damage to normal cells (10, 14, 20). Collectively, this evidence suggested that MASTL was an attractive target for selective anticancer treatment and as a therapeutic combination with radiotherapy.

The drug discovery process has changed greatly by the application of computer-aided drug discovery-design methods such as *in silico* high-throughput screening of candidate compounds based on the structural analysis of protein-ligand binding interactions (21, 22). MASTL is structurally classified as a member of the AGC kinase family (23). Recently, the protein structure of the kinase domain of MASTL was solved and a first-line inhibitor against MASTL, GKI-1, was reported (24). In addition, two compounds were predicted as potential MASTL inhibitors (25). However, it was unclear whether these compounds had antitumor or radiosensitizer activity in human cancer cells. In this study, we have identified and validated a new MASTL inhibitor, named MKI-1 (MASTL Kinase Inhibitor-1), through *in silico* screening and *in vitro* analysis. We demonstrated that MKI-1 exerted antitumor and radiosensitizer activities through increased PP2A activity and c-Myc destabilization *in vitro* and *in vivo*. Moreover, the regulatory activity of MKI-1 on MASTL-PP2A was validated in a mouse oocyte model. Hence, our data have provided a new-line inhibitor of MASTL with antitumor and radiosensitizing activities in breast cancer.

## Materials and Methods

### Cell Culture and Treatment

Cell lines were purchased from American Type Culture Collection (ATCC; Manassas, VA, United States) and authenticated by their karyotype, image, and a detailed analysis of gene expression. MCF7 and T47D cells were maintained in DMEM (Corning, NY, United States) and BT549 cells were cultured in RPMI 1640 (Welgene, Daegu, South Korea); both media were supplemented with 10% fetal bovine serum (Corning) and 1% penicillin/streptomycin. Cells were maintained in a humidified 5% CO_2_ incubator at 37°C. MCF10A cells were maintained in DMEM/F12 (Invitrogen, CA, United States) supplemented with 5% heat-inactivated horse serum (Invitrogen), 1% penicillin/streptomycin, 20 ng/mL EGF (Peprotech, London, United Kingdom), 0.5 mg/mL hydrocortisone (Sigma-Aldrich, MO, United States), 100 ng/mL cholera toxin (Sigma-Aldrich), and 10 μg insulin (Sigma-Aldrich). The radioresistant CD44^*high*^/CD24^*low*^ MCF7 cells were established as reported (26, 27). The cells were irradiated by using a ^137^cesium (Cs) gamma ray source (Atomic Energy of Canada Ltd., Mississauga, ON, Canada) at a dosage of 3.81 Gy/min.

### *In silico* Screening

The X-ray crystal structure of MASTL was solved with staurosporine (5LOH.pdb) (24). Based on this structure, *in silico* screening was performed with docking simulation. The Glide docking tool in MAESTRO (Schrödinger LLC, NY, United States), which uses a receptor grid-based method to define the ligand-binding site, was used for docking with a commercially available library that included 200,000 compounds (ChemBridge Corp., CA, United States) (28). The Glide tool filters molecules using XP (extra precision) modes (29). The docking score (Glide score) and visual inspection were used to guide candidate compound selection. The selection criteria for *in silico* hits were established by using the Glide score. The Glide score is a scoring function that separates compounds that do not bind to a protein and those that bind strongly after the docking simulation (29). After the docking simulation, 300 compounds with the highest score were shortlisted and the final 40 candidate compounds were selected by visual inspection. The RMSD cut-off range was less than 0.5 Å, which is the default value in MAESTRO program.

### Chemicals and Treatments

The 40 compounds required for *in silico* screening were purchased from ChemBridge Corporation. AT13148 (Selleck Chemicals, TX, United States), GKI-1, which was synthesized by Korea Research Institute of Chemical Technology (Daejeon, South Korea), and MKI-1 (Cat. 9335496; ChemBridge Corp.) were treated at the indicated concentrations. In particular, for a long-term treatment such as clonogenic, mammosphere formation assays, and 3D culture, 1 μM AT13148 (Selleck Chemicals), 10 μM GKI-1 (KRICT), and 10 μM MKI-1 (ChemBridge Corp.) were used to reduce the toxicity compared to cell viability assay. In clonogenic assays for the radiation sensitivity assay, 0.5 μM AT13148, 7.5 μM GKI-1, and 7.5 μM. MKI-1 were used in order to observe the combination effect between compounds and radiation.

### Solubility and Purity of the Candidate Compound MKI-1

Kinetic solubility of the candidate compound MIK-1 was measured using nephelometry in the Korea Research Institute of Chemical Technology. Kinetic solubility of the candidate compound MIK-1 was 203.3 ± 0.9 μM in the 5% DMSO/H_2_O solvent system.

Prior to biological testing, final compounds were confirmed to be >95% pure by UPLC chromatography using a Waters ACQUITY H-class system fitted with a C18 reversed-phase column (ACQUITY UPLC BEH C18: 2.1 mm × 50 mm, Part No. 186002350).

### Cell Viability Assays

Cell viability was determined by using the WST-8 assay (Cyto X cell viability assay kit; LPS solution, Daejeon, South Korea) as recommended by the manufacturer.

### RNA Interference

The following constructs were used for RNA interference: MASTL, 5′-GAAUGAACUUGCAUAAUUAUU-3′, PP2A-B55-α, PP2A-B55-δ, and PP2A-B56-γ (PP2A subunit siRNAs were purchased from Santa Cruz Biotechnology). Non-silencing siRNA (Bioneer, Daejeon, South Korea) was used as a negative control. Transfection of siRNAs (20 nM) was performed using G-fectin (Genolution, Seoul, South Korea) as recommended by the manufacturer.

### *In vitro* Kinase Assay

For the *in vitro* kinase assays, recombinant GST-tagged-MASTL (Thermo Fisher Scientific, MA, United States) and His-tagged-ENSA (Sino Biological Inc., Beijing, China) or the immunoprecipitated MASTL from the lysate of MCF7 cells using anti-MASTL antibody (AP7147d; Abgent, CA, United States) were reacted in kinase buffer [100 mM Tris–Hcl (pH 7.5), 30 mM MgCl_2_, 2 mM DTT, 1 mM EDTA, 10 μM ultra-pure ATP (Promega, WI, United States)] for 30 min at 30°C. The reactions were stopped with SDS-loading buffer, and immunoblotting analysis was used to detect phosphorylated ENSA.

### ADP-Glo Luminescence-Kinase Assay

ADP-Glo kinase assays were performed as recommended by the manufacturer (Promega).

### Clonogenic and Sphere Formation Assay

Cell survival after irradiation was determined by using a clonogenic assay (27). The sphere formation assay was performed as previously reported (14, 26).

### Three-Dimensional Culture

Three-dimensional culture was performed as recommended by the manufacturer (TheWell Bioscience, NJ, United States).

### Quantitative Real-Time PCR

qRT-PCR was performed as previously described (27). The following primer sequences were used: β-actin forward, 5′-CATGTACGTTGCTATCCAGGC-3′; β-actin reverse, 5′-CTC CTTAATGTCACGCACGAT-3′; survivin forward, 5′-GGCCC AGTGTTTCTTCTGCTTCTGCTTC-3′; reverse, 5′-GCACTT TCTCCGCAGTTTCCTC-3′; cyclin D1 forward, 5′-ATGC CAACCTCCTCAACGAC-3′; reverse, 5′-GGCTCTTTTTCA CGGGCTCC-3′; cyclin B1 forward, 5′-ACTGTCTCCA TTATTGATCG-3′; reverse, 5′-TGTCCATTCACCATTATC-3′; β-actin was used as an internal control.

### Western Blotting Analysis

Western blotting was performed as described (27, 30). The following antibodies were used: rabbit polyclonal antibodies against MASTL (Abgent); phospho-ENSA (Ser67)/ARPP19 (Ser62), ENSA, cleaved PARP (Asp214), AKT, phospho-AKT (ser473), phospho-GSK-3α/β (Ser21/9), phospho-p70S6K (Thr389), phospho-Y15-Cdk1, phospho-Chk2 (Thr68) (Cell Signaling Technology), p70S6K, and Cdk1 (Santa Cruz Biotechnology); rabbit monoclonal antibody against phospho-c-Myc (Ser 62) (Abcam, United Kingdom); mouse monoclonal antibody against caspase-2 (Cell Signaling Technology), His-probe, and c-Myc (Santa Cruz Biotechnology); and a mouse polyclonal antibody against β-actin (Santa Cruz Biotechnology).

### Immunofluorescence

Immunofluorescence analysis was performed as recommended by the manufacturer (30, 31).

### PP2A Activity Assay

A PP2A activity assay was performed as previously described (30), and the PP2A phosphatase assay followed the manufacturer’s protocol (RediPlate 96 EnzChek serine/threonine phosphatase assay kit; Invitrogen).

### Xenograft Studies

Five-week-old female BALB/c nude mice were purchased from ORIENT Bio (Seongnam, South Korea) and maintained under aseptic conditions for 1 week. BT549 cells (5 × 10^6^ cells) were implanted into the inguinal mammary fat pad. Mice were randomized into four groups, each containing five mice: the vesicle (Ctrl; 10% DMSO + 1% Tween 20 + 89% saline); 6 Gy; MKI-1; and 6 Gy with MKI-1. MKI-1 was dissolved in 10% DMSO (Sigma-Aldrich), 1% Tween 20 (Sigma-Aldrich), and 89% saline (Sigma-Aldrich), and the animals received MKI-1 (50 mg/kg) twice per week by intraperitoneal (i.p.) injection. MKI-1 or 6 Gy was administered to mice bearing tumors of approximately 50 or 100 mm^3^, respectively. Tumor volumes were measured three times per week by using a caliper. The mice were sacrificed 31 days after the tumor cells were seeded, after which the subcutaneous tumors were excised and weighed. Animal care and treatment were followed by institutional guidelines. All testing was carried out at the animal laboratory of KIRAMS after ethical approval (#2018-0062).

### Mouse Oocyte Culture and Treatment

Mouse oocytes at the germinal vesicle (GV) stage were recovered from ovaries of 3-week-old CD-1 female mice (Koatech) that had been administered 5 IU of pregnant mare’s serum gonadotrophin (Sigma-Aldrich). Isolated oocytes were placed in M2 medium (Zenith Bio) supplemented with 100 μM 3-isobutyl-1-methylxanthine (IBMX; Sigma-Aldrich) to maintain GV arrest. To resume meiosis, oocytes were placed in IBMX-free medium in a 5% CO_2_ atmosphere at 37°C. Oocytes were treated with the indicated concentration of chemicals or an equivalent amount of DMSO.

### Statistical Analysis

The two-tailed Student’s *t*-test was performed to analyze statistical differences between groups. *P*-values of less than 0.05 were considered statistically significant. Statistical analyses were computed by using Excel and XLSTAT software.

## Results

### Identification of MASTL Inhibitors Using *in silico* Screening and *in vitro* Analysis

**FIGURE 1:**
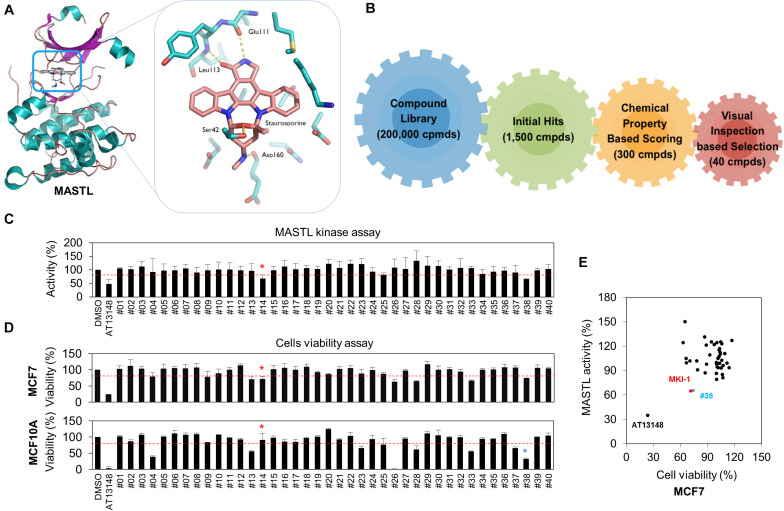
Identification of MASTL inhibitor candidates by *in silico* screening and *in vitro* analysis. **(A,B)** The docking model of STA and the MASTL kinase domain **(A)** and a schematic summary of *in silico* screening **(B)**. **(C)** MASTL activities were determined by the ADP-Glo kinase assay. The arbitrary threshold was set at 30% viability and 30% luciferase inhibition. **(D)** MCF7 and MCF10A cells were treated with DMSO (Ctrl), 20 μM AT13148, and 20 μM candidate compounds (#1–#40) for 72 h. Cell viability was measured by using the WST-8 assay. **(E)** Compounds that inhibited cell viability and luciferase activity by 30% or more were selected as candidates. The cut-off value of 30% reduction of viability was determined by the minimum inhibition rate of viability or activity in MASTL-depleted MCF7 breast cancer cells. Red line indicates cut-off line. The data are presented as the mean ± standard deviation of three independent experiments. Asterisk indicates the candidate compound.

The crystal structure of the kinase domain of MASTL was solved with staurosporine (STA), a well-known kinase inhibitor (24), and *in silico* screening was performed based on the binding model between STA and the MASTL kinase domain. Like most kinase inhibitors, STA forms hydrogen-bonding interactions at the hinge-region of MASTL with the backbone of leucine 113 (L113) (Figure 1A). Based on these interactions, we performed a docking simulation with a commercially available library, and further determined the docking score and visual inspection from the initial hits. Finally, 40 candidate compounds were selected (Figure 1B). The range of docking score for 40 compounds was from −9.2 to −8.1, indicating a good binding capacity. Next, a luminescence-based kinase assay for 40 candidate compounds was performed using recombinant MASTL (Figure 1C) and the viability of MCF7 breast cancer cells and MCF10A mammary normal cells was measured (Figure 1D), because MASTL candidate inhibitors are considered to exert antitumor activity with low toxicity to normal cells such as MCF10A (10, 14, 32). In these analyses, AT13148, a multi-AGC kinase inhibitor (33), was used as the positive control. Of the initial two hit compounds, compound #14 [N-1H-benzimidazol-2-yl-3-(1H-pyrrol-1-yl) benzamide] was finally selected as a potential candidate MASTL inhibitor, because compound #38 was cytotoxic to MCF10A normal mammary cells (Figure 1E). Collectively, our data indicated that N-1H-benzimidazol-2-yl-3-(1H-pyrrol-1-yl), a benzamide compound, named as MKI-1, was a new candidate MASTL inhibitor in breast cancer cells.

### *In vitro* Validation of MKI-1 as a Novel MASTL Inhibitor

**FIGURE 2:**
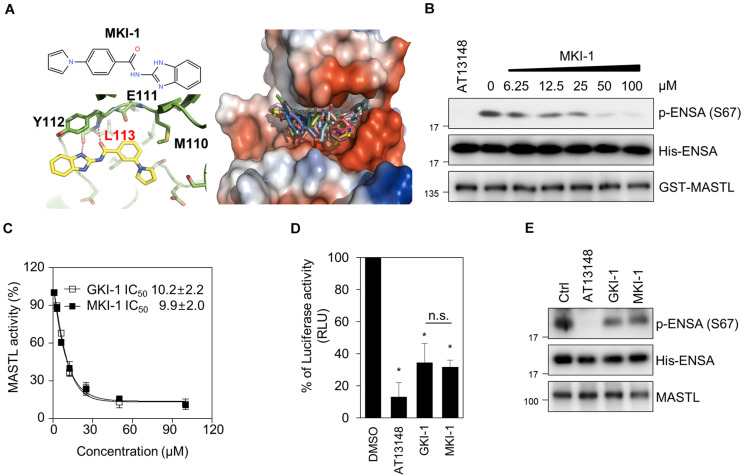
MKI-1 inhibits MASTL activity *in vitro*. **(A)** Chemical structure of MKI-1 (left; upper panel). The docking models of MKI-1 and the MASTL kinase domain (left; lower panel and right). **(B,C)** The *in vitro* kinase assay of recombinant MASTL with ENSA as a substrate was performed with Ctrl (DMSO), 20 μM AT13148, and the indicated doses of MKI-1 **(B)** or the indicated doses of GKI-1 **(C)**. The *in vitro* kinase assay was analyzed by immunoblotting with the indicated antibodies **(B)**. The percentage kinase activity was measured by using the ADP-Glo kinase assay to determine the IC_50_ **(C)**. The percentage kinase activity was normalized to the control, and the smallest and largest values were defined and plotted as 0 and 100% kinase activity, respectively. **(D)** MASTL activities were determined by the ADP-Glo kinase assay. **(E)** The *in vitro* kinase assay of immunoprecipitated MASTL from the lysate of mitotic MCF7 cells, treated with colcemide (80 ng/mL) for 16 h, was performed with DMSO (Ctrl), 20 μM AT13148, 20 μM GKI-1, and 20 μM MKI-1, and was then analyzed by immunoblotting with the indicated antibodies. The data represent typical results and are presented as the mean ± standard deviation of three independent experiments. *P < 0.01.

The L113 amino acid on MASTL was predicted to play a key role in the binding of MKI-1. The oxygen atom of L113 forms a strong hydrogen bond with the N–H group of the benzimidazole moiety and the amine functional group of L113 forms another strong hydrogen bond with the carbonyl oxygen atom of MKI-1 (Figure 2A, left panel). The docking model predicted that MKI-1 was bound to L113, which was presented in the kinase domain of MASTL by two hydrogen bonds, allowing the correct position for entry into the binding pocket of MASTL. The phenylpyrrolidine moiety of MKI-1, a lipophilic functional group, was tilted against methionine 110 in the kinase pocket of MASTL, and did not interact with the kinase pocket (Figure 2A, right panel). MKI-1 inhibited the kinase activity of MASTL in a dose-dependent manner (Figure 2B). A luminescence-based kinase assay indicated that the IC_50_ of MKI-1 was similar to that of GKI, a first-line inhibitor of MASTL *in vitro* (24), in an *in vitro* kinase assay (Figure 2C). In addition, we confirmed that MKI-1 also inhibited the kinase activity of endogenous MASTL, as determined by an immunoprecipitation kinase assay (Figures 2D,E). In these assays, AT13148 and/or GKI-1 were used as positive controls. Collectively, our data indicated that MKI-1 inhibits the kinase activity of MASTL *in vitro*.

### MKI-1 Inhibits MASTL in Breast Cancer Cells

**FIGURE 3:**
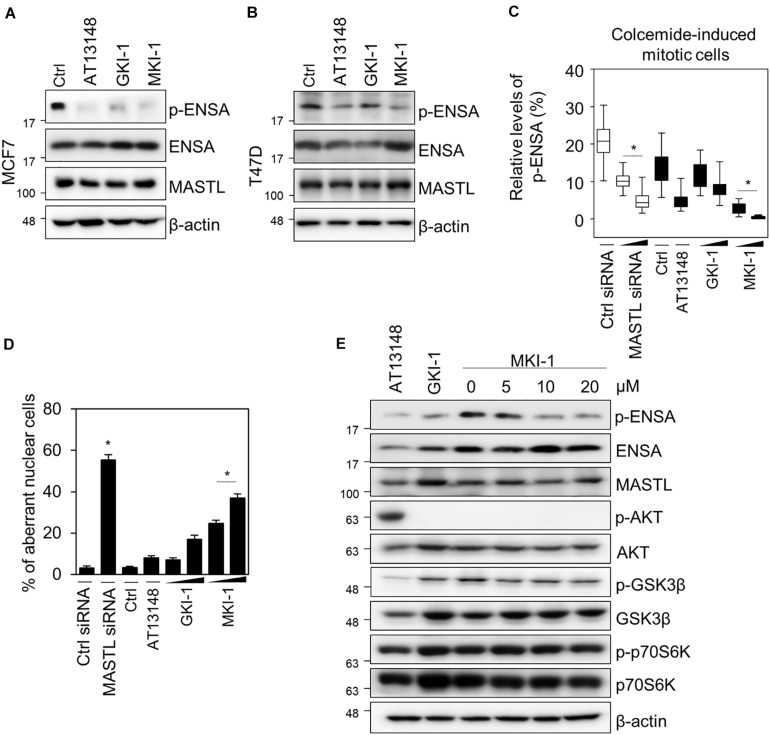
MKI-1 inhibits the activity of MASTL in breast cancer cells. **(A,B)** MCF7 and T47D cells were treated with DMSO (Ctrl), 5 μM AT13148, 15 μM GKI-1, and 15 μM MKI-1 for 20 h. The cell lysates were analyzed by immunoblotting with the indicated antibodies; β-actin was used as the loading control. **(C,D)** MCF7 cells transfected with either control siRNA or MASTL siRNA (20 or 40 nM) for 24 h were arrested in mitosis using colcemide (80 ng/mL) for 16 h or MCF7 cells with colcemide were treated with Ctrl (DMSO), 5 μM AT13148, GKI-1 (15 or 30 μM), and MKI-1 (15 or 30 μM) for 16 h. Immunofluorescence staining was performed using anti-phospho(Ser67) ENSA. The intensities of phospho-ENSA or aberrant nuclear cells such as nuclei with irregular shapes or fragmented nuclei were scanned and determined by using the IN Cell Analyzer HCA System. At least 17 images, each image contains more than 100 cells, were analyzed from each group by IN-Cell analyzer. **(E)** MCF7 cells were treated with 5 μM AT13148 for 24 h, 15 μM GKI-1 for 24 h, and MKI-1 (at the indicated doses) for 24 h. Cell lysates were analyzed by immunoblotting with the indicated antibodies; β-actin was used as the loading control. The data represent typical results and are presented as the mean ± standard deviation of three independent experiments. *P < 0.01.

To confirm that MKI-1 inhibited MASTL in breast cancer cells, we first determined whether MKI-1 inhibited the phosphorylation of ENSA in two breast cancer cell lines, MCF7 and T47D, which cells expresses high MASTL (12–14). As seen in the *in vitro* kinase assay, MKI-1 inhibited the phosphorylation of ENSA in MCF7 and T47D cells (Figures 3A,B). In addition, we also examined whether MKI-1 inhibited the phosphorylation of ENSA in colcemid-induced mitotic MCF7 cells. Our immunofluorescence data indicated that MKI-1 significantly inhibited the phosphorylation of ENSA in mitotic cells (Figure 3C and Supplementary Figure 1A) compared with the positive controls of siRNA-mediated MASTL depletion, AT13148, and GKI-1. We also determined whether MKI-1 increased aberrant nuclei in MCF7 cells, as we had previously observed that MASTL depletion increased aberrant nuclei such as nuclei with irregular shapes or fragmented nuclei in breast cancer cells (14). Similar to the result of increased aberrant nuclei in the MASTL-depleted cells, MKI-1, but not AT13149, increased aberrant nuclei in MCF7 cells (Figure 3D and Supplementary Figure 1B). Furthermore, we found that MKI-1 did not modulate phosphorylated AKT, GSK-3β, and p70S6K in a dose-dependent manner in MCF7 cells; in contrast, AT13148 clearly increased AKT phosphorylation, but decreased phosphorylated GSK-3β and p70S6K (Figure 3E). Thus, our results suggested that MKI-1 inhibited MASTL in breast cancer cells.

### MKI-1 Inhibits Various Oncogenic Properties of Breast Cancer Cells but Showed Much Weaker Effects on the Viability of Normal Breast Cells

**FIGURE 4:**
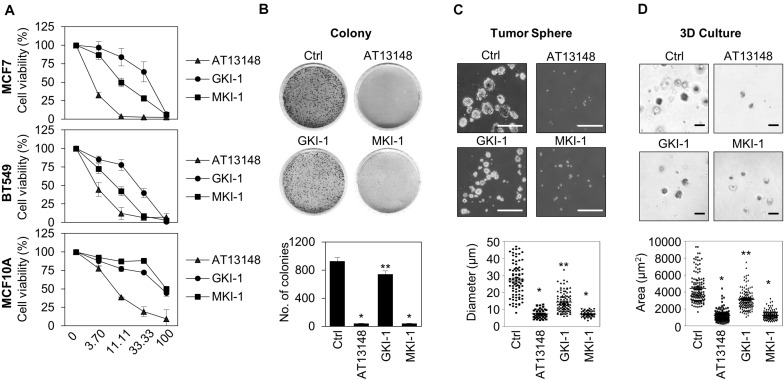
MKI-1 inhibits various oncogenic properties of MCF7 cells and does not affect the viability of MCF10A cells. **(A)** MCF7, BT549, and MCF10A cells were treated with AT13148, GKI-1, and MKI-1 with the serial dilutions from 100 μM for 72 h. The cell viabilities were determined by using the WST-8 assay. **(B–D)** MCF7 cells were treated with DMSO (Ctrl), 1 μM AT13148, 10 μM GKI-1, and 10 μM MKI-1. Colony formation was determined by using the colony formation assay **(B)**. Representative images (upper panel) and the quantification (lower panel) of colony formation of MCF7 cells. **(C)** Mammosphere formation was determined by using the sphere formation assay. **(D)** Spheroid formation was determined by using the 3D culture assay. Representative images (upper panel) and quantification (lower panel) of tumor sphere formation of MCF7 cells (>110 sphere colonies per data point). Scale bars = 100 μm. The data represent typical results and are presented as the mean ± standard deviation of three independent experiments. **P < 0.05 and *P < 0.01.

As we showed that MASTL depletion reduced the oncogenic properties of breast cancer cells and did not affect normal breast cells (14), we examined the antitumor activity of MKI-1 in breast cancer cells through various analyses, including: cell viability, clonogenic, and mammosphere formation assays, and 3D culture. MKI-1 was more active in MCF7 and BT549 breast cancer cells than in MCF10A normal breast cells (Figure 4A). In addition, MKI-1 clearly inhibited the colony and mammosphere formation of MCF7 cells, whereas GKI-1 slightly reduced these activities (Figures 4B–D). AT13148 had the most potent antitumor activity, but it was cytotoxic to healthy breast cells (Figure 4A). Therefore, our results suggested that MKI-1 inhibited the oncogenic properties of breast cancer cells, with low toxicity to normal breast cells.

### MKI-1 Reduces Tumor Growth and Enhanced the Radiosensitivity of Breast Cancer Cells

**FIGURE 5:**
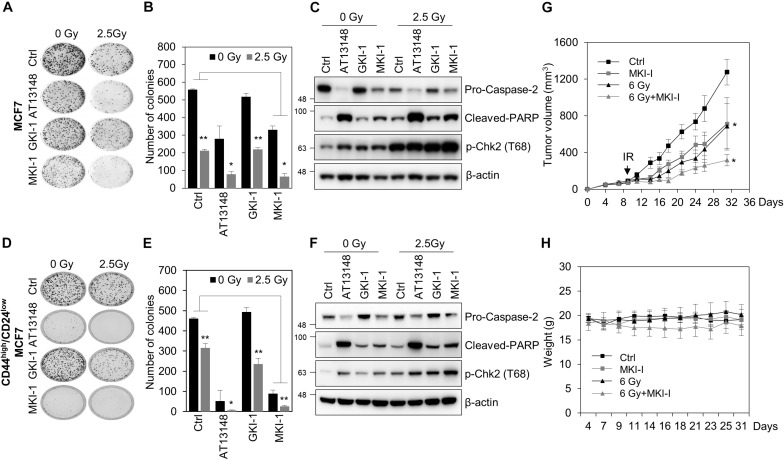
MKI-1 reduced tumor growth and radiosensitivity of breast cancer cells. **(A–F)** MCF7 cells **(A–C)** or CD44^*high*^/CD24^*low*^ MCF7 cells **(D–F)** were treated with DMSO (Ctrl), 0.5 μM AT13148, 7.5 μM GKI-1, and 7.5 μM MKI-1 without or with 2.5 Gy radiation for 14 days **(A,B,D,E)** or 24 h **(C,F)**. Colony formation was determined by using the colony formation assay. Representative images **(A,D)** and the quantification **(B,E)** of colony formation of MCF7 cells treated with indicated conditions. Cell lysates were analyzed by immunoblotting with the indicated antibodies **(C,F)**. **(G,H)** Female BALB/c nude mice were subcutaneously injected with 5 × 10^6^ BT549 cells, and treated with DMSO (Ctrl), MKI-1 (50 mg/kg two times per week), 6 Gy, or 6 Gy with MKI-1. MKI-1 was treated for 6 h prior to irradiation. The tumor volume and weight were calculated over time, as indicated, in each treatment groups (*n* = 5) **(G,H)**. The data represent the typical results and are presented as the mean ± standard deviation of three independent experiments. **P < 0.05 and *P < 0.01.

As MASTL is a candidate target for radiosensitization in non-small cell lung cancer cells (19) and MASTL depletion inhibits the radioresistant breast cancer stem cells (BCSCs) through caspase-2 activation, which is a major caspase in the mitotic catastrophe in response to DNA damage (14), we examined the effects of MKI-1 on the radiosensitivity of breast cancer cells. MKI-1 clearly decreased colony formation in MCF7 cells in response to irradiation (Figures 5A,B), with increases in cleaved-PARP and phosphorylated Chk2, and a decrease in procaspase-2 (Figure 5C), which was consistent with the effects of MASTL depletion (14). The radiosensitizer activity of MKI-1 was further validated by using radioresistant BCSCs (26). BCSCs were sorted from MCF7 cells by using CD44^*high*^/CD24^*low*^, a marker for BCSCs (34). We have previously demonstrated that CD44^*high*^/CD24^*low*^ MCF7 cells are BCSCs with a radioresistant phenotype (26). MKI-1 also reduced colony formation of BCSCs in response to irradiation (Figures 5D,E), with increases in cleaved PARP and phosphorylated Chk2, and a decrease in procaspase-2 (Figure 5F). Next, the *in vivo* antitumor and radiosensitizer activity of MKI-1 was further examined by using the BT549 xenograft mouse model, since a previous report showed that MASTL inhibition is more sensitive to triple-negative breast cancer cells including BT549 (12). Consistent with the *in vitro* data, MKI-1 treatment reduced tumor growth and enhanced the radiosensitivity of BT549 xenograft model in response to 6 Gy irradiation compared with the control group (Figure 5G), with no notable changes in body weight (Figure 5H), suggesting the absence of gross toxicity in the treated mice. Therefore, these results suggested that MKI-1 inhibits tumor growth and enhances the radiosensitivity of breast cancer cells.

### MKI-1 Activates PP2A and Decreases c-Myc Stability in Breast Cancer Cells

**FIGURE 6:**
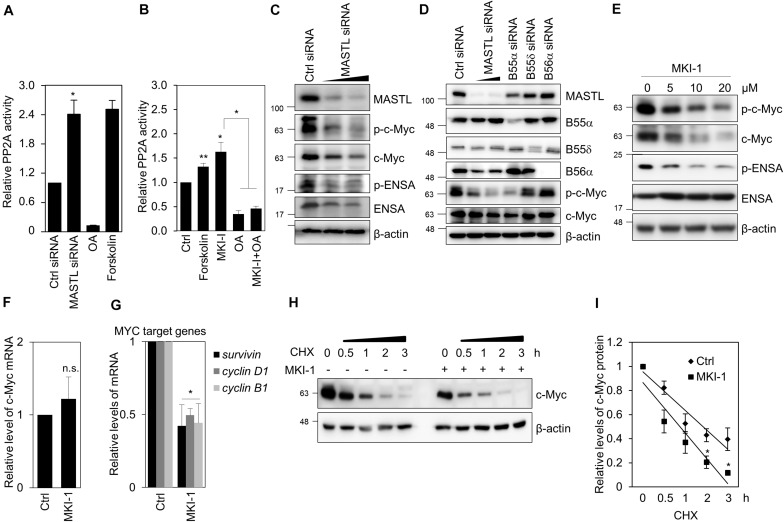
MKI-1 reduced c-Myc stability through PP2A activation in MCF7 cells. **(A,C,D)** MCF7 cells transfected with either control siRNA, MASTL siRNA, B55α siRNA, B55δ siRNA, or B56α siRNA for 36 h. **(B)** MCF7 cells were treated with DMSO (Ctrl), 40 μM forskolin, 20 μM MKI-1, 50 nM OA, or 50 nM OA with 20 μM MKI-1 for 16 h. PP2A proteins were immunoprecipitated using anti-PP2A-Cα/β antibody and analyzed for PP2A activity **(A,B)**. **(E–G)** MCF7 cells were treated with DMSO (Ctrl) or the indicated doses of MKI-1 **(E)** or 10 μM MKI-1 **(F,G)** for 24 h. Cell lysates were analyzed by immunoblotting with the indicated antibodies **(C–E)** or qRT-PCR **(F,G)**; β-actin was used as the loading control. **(H,I)** MCF7 cells were treated without or with MKI-1 for 24 h, and then cycloheximide (100 μg/mL) was added for the indicated time periods. The protein expression of c-Myc was determined by immunoblotting **(H)**. c-Myc expression was quantified by using Image Quant and normalized using β-actin as internal control **(I)**. The data represent the typical results and are presented as the mean ± standard deviation of three independent experiments. **P < 0.05 and *P < 0.01. n.s., not significant.

As MASTL inhibits PP2A activity in various models (6–8), including cancer cells (12, 14), we examined whether MKI-1 regulated PP2A activity in breast cancer cells. Forskolin and Okadaic acid (OA) were the positive and negative controls for PP2A activity, respectively (30). First, we confirmed that MASTL depletion increased PP2A activity in MCF7 cells (Figure 6A). Similarly, MKI-1 increased PP2A activity and this activation was inhibited by OA treatment (Figure 6B), suggesting that MKI-1 has the capacity to activate PP2A. Next, we examined the possibility that MASTL inhibition modulated c-Myc protein, as it is well established that PP2A activity is negatively associated with c-Myc (35) and that MASTL regulates the activity of PP2A-B55α/δ (6, 8, 12). We found that MASTL depletion decreased c-Myc protein levels (Figure 6C), and that PP2A-B55α depletion more strongly decreased both serine 62-phosphorylation of c-Myc and total c-Myc compared with PP2A-B55δ and PP2A-B56α in MCF7 cells (Figure 6D), suggesting that MASTL may regulate c-Myc via PP2A-B55α in breast cancer cells. Consistently, MKI-1 clearly reduced both serine 62-phosphorylation of c-Myc and total c-Myc, with a decrease in ENSA phosphorylation (Figure 6E). However, MKI-1 did not significantly alter the mRNA expression of c-Myc (Figure 6F), but inhibited the Myc target genes, such as survivin, cyclin D1, and cyclin B1 (Figure 6G), which implied that MKI-1-mediated c-Myc inhibition was regulated at the post-transcriptional level. Indeed, we found that MKI-1 reduced the stability of c-Myc protein after cycloheximide treatment (Figures 6H,I). These results suggested that MKI-1 was able to reduce c-Myc stability through PP2A activation in breast cancer cells.

### MKI-1 Inhibits Meiotic Maturation of Mouse Oocytes Dependent on PP2A

**FIGURE 7:**
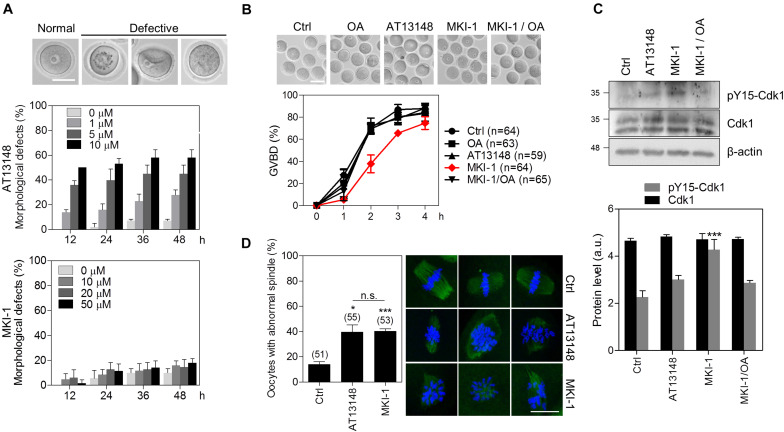
MKI-1 inhibits meiotic resumption and spindle formation in mouse oocytes. **(A)** GV oocytes were treated with the indicated doses of AT13148 or MKI-1 and then morphological defects were determined for up to 48 h. Representative images of normal and defective oocytes are shown. Scale bars = 50 μm. **(B)** GV oocytes were treated with DMSO (Ctrl), 100 nM OA, 1 μM AT13148, 10 μM MKI-1, or 10 μM MKI-1 with 100 nM OA. After GV arrest was released by washing out IBMX, the GVBD rate was measured for 4 h. The number of oocytes analyzed is shown in parenthesis. Representative images of oocytes taken 2 h after IBMX release are shown. Scale bars = 100 μm. **(C)** Oocyte lysates at 2 h after IBMX release were analyzed by immunoblotting for the pY15-Cdk1 antibody. Total β-actin was used as the loading control. For western blotting, the samples were collected and loaded into separating gel to generate replicate blots. **(D)** Oocytes treated with DMSO (Ctrl), 1 μM AT13148 or 10 μM MKI-1 were cultured for 18 h in the absence of IBMX. Immunofluorescence staining was performed using anti-α-tubulin (green). DAPI was used to stain the nucleus (blue). The data are presented as the mean + SEM from three independent experiments with representative images of the spindle. The number of oocytes analyzed is shown above the bars. Scale bars = 20 μm. ***P < 0.001 and *P < 0.01. n.s., not significant.

As the function of MASTL has been well studied by using oocytes as a model system (9, 36, 37), we validated MKI-1 activity in mouse oocytes. First, we compared the cytotoxicity of MKI-1 and AT13148 in mouse oocytes. MKI-1 did not cause any morphological defects in GV oocytes, whereas AT13148 induced severe morphological defects (Figure 7A); this implied that MKI-1 was not cytotoxic to oocytes. Next, the effects of MKI-1 treatment on the meiotic resumption of oocytes were examined. Consistent with previous findings that MASTL depletion caused GV arrest or delayed GV breakdown (GVBD) through inhibition of the timely activation of CDK1 (9, 37), we observed that MKI-1 treatment delayed GVBD and decreased CDK1 activity, whereas AT13148 did not affect either GVBD or CDK1 activity (Figures 7B,C). Importantly, this phenotype was rescued by OA treatment, which suggested that MKI-1 activity was dependent on PP2A activation during the meiotic resumption of mouse oocytes. Moreover, MKI-1 treatment induced spindle defects in mouse oocytes (Figure 7D), similar to the phenotype observed in MASTL-depleted oocytes (9, 37). Thus, we have validated the MKI-1-mediated inhibition of PP2A by using mouse oocytes.

## Discussion

Recent studies suggested MASTL was an attractive target for anticancer treatment owing to its regulation of various oncogenic properties, including cellular transformation, metastasis, chromosomal instability, and the DNA damage response (11, 13, 16); however, a MASTL inhibitor with antitumor activity has not yet been reported. Here, we identified and evaluated the compound MKI-1 as a new-line MASTL inhibitor with antitumor activity against breast cancer cells and minimal effects on normal breast cells. We also showed that MKI-1 exerted radiosensitizer activity *in vitro* and *in vivo*. Interestingly, we found that MKI-1 reduced c-Myc stability through PP2A activation. Furthermore, the activity of MKI-1 in the regulation of PP2A was validated by using mouse oocytes. Therefore, our study provides evidence that MKI-1 is a new inhibitor of MASTL with antitumor and radiosensitizer activities resulting from PP2A activation in breast cancer.

Two groups recently reported candidate compounds for MASTL inhibitors (24, 25). Ocasio et al. (24) showed that GKI-1 inhibited the kinase activity of MASTL *in vitro* (IC_50_ = 5–9 μM) and the phosphorylation of ENSA in HeLa cells, which was similar to our observations. Ammarah et al. (25) provided two potential MASTL inhibitors that were predicted to interact with the kinase domain of MASTL. However, it was not reported whether these compounds had antitumor activity against cancer cells *in vitro* or *in vivo*. Our data clearly showed that MKI-1 had potent antitumor activity in the *in vitro* and *in vivo* models. In addition, we observed that MKI-1 inhibited ENSA phosphorylation at 6 h and it did not modulate the phosphorylation levels of other AGC kinases, including AKT, p70S6K, and GSK-3β, in breast cancer cells, whereas AT13148 and GKI-1 modulated these kinases (Supplementary Figure 2), suggesting that MKI-1 had a greater capacity for MASTL inhibition than other inhibitors. However, the active concentration of MKI-1 for MASTL inhibition was relatively high, approximately 10-fold greater than that of the multiple AGC kinase inhibitor AT13148; therefore, further study of the active derivatives of MKI-1 is required to increase the selectivity and antitumor activity of targeting MASTL.

Our data showed that MKI-1 had low toxicity in normal human breast cells. Several studies showed that MASTL is highly overexpressed in multiple cancers, including breast cancer, compared with normal tissues (11–14). In addition, our previous study showed that MASTL depletion did not affect the viability of healthy breast cells with low expression of MASTL (14). Similarly, MASTL depletion did not affect the viability of various normal cell lines, including oral keratinocyte cells, dermal fibroblast cells, and umbilical vein endothelial cells (10, 14). In addition, our data showed that MKI-1 inhibited various oncogenic properties, such as cancer stemness, which phenotype was consistent to the effect of MASTL depletion in breast cancer cells (14). Therefore, these results support our conclusion that MKI-1 reduces the oncogenic properties of breast cancer cells less affecting normal breast cells.

Microtubule-associated serine/threonine kinase-like was reported as a potential target for radiosensitization in non-small cell lung cancer cells and breast cancer cells (19), and is known to regulate recovery from the S/G_2_ DNA damage checkpoint (16, 17). In addition, we previously showed that MASTL depletion inhibited radioresistant BCSCs (14), a key mediator of the radioresistance of tumor cells (38), indicating that MASTL inhibitors may be used as a radiosensitizer. Indeed, our data showed that MKI-1 was a potential radiosensitizer in breast cancer *in vitro* and *in vivo*. MKI-1, combined with radiation, reduced colony formation and breast cancer stemness with the activation of Chk2 and caspase-2, suggesting that MKI-1 may have the capacity to inhibit the intrinsic radioresistance of breast cancer cells through modulation of the mitotic DNA damage response, consequently leading to mitotic cell death following irradiation. Therefore, it is likely that MASTL inhibitors, such as MKI-1, are useful drugs for both anticancer treatment and as a potential radiosensitizer in breast cancer.

Our data showed that MKI-1 activated PP2A and reduced c-Myc stability in breast cancer cells. MASTL has the capacity to promote cellular transformation (11), whereas most mitotic kinases, including Plk1, Aurora A/B, and Nek2, are unable to induce cellular transformation in human malignancies (1). These suggested that MASTL-mediated tumor progression was dependent on the tumor-suppressive action of PP2A, as PP2A dephosphorylates many oncogenic factors involved in the process of cellular transformation, such as c-Myc (35). It is well known that the activation of PP2A dephosphorylates the serine-62 phosphorylation on c-Myc and thereby promotes c-Myc proteolysis via the ubiquitin-proteasome pathway (35, 39). Recently, two independent groups showed that MASTL inhibited PP2A activity in breast cancer cells (12, 14), suggesting that the oncogenic role of MASTL is associated with PP2A inhibition. Another report showed that MASTL inhibition reduced c-Myc and its target genes, such as survivin and Bcl-xl, in colon cancer cells (20). In our study, we showed that PP2A-B55α, a key downstream target of MASTL (5, 8, 12, 14), reduced c-Myc phosphorylation and protein level, suggesting that c-Myc may be regulated by PP2A-B55 in breast cancer cells. Similarly, reports showed that PP2A-B55α inhibited the stability of c-Myc in breast cancer cells (40). Therefore, our data suggested that the antitumor activity of MKI-1 was exerted through PP2A-mediated c-Myc regulation in breast cancer cells.

In this study, we utilized mouse oocytes as a model system to validate MKI-1 activity. Our data showed that MKI-1 delayed GVBD, and decreased CDK1 activity that was reversed by OA treatment. We also found that MKI-1 inhibited spindle formation during meiotic maturation. Consistent with our results, the function of MASTL in meiotic resumption and spindle formation has been well demonstrated in *Xenopus*, *Drosophila*, mouse, and porcine oocytes (9, 36, 37, 41, 42). Moreover, the meiotic progression driven by MASTL is known to be associated with PP2A activity in all oocyte model systems (9, 36, 37, 41–43). Therefore, our data in mouse oocytes have confirmed that MKI-1 is a regulator of PP2A activity.

## Conclusion

In conclusion, we identified and functionally characterized a new MASTL inhibitor, MKI-1, that reactivates PP2A by the inhibition of MASTL-ENSA. We showed that MKI-1 exerted antitumor and radiosensitizer activities through PP2A-mediated c-Myc inhibition in breast cancer models, which suggested that MASTL targeting may be associated with the MASTL-PP2A-c-Myc axis in breast cancer.

## Data Availability Statement

Publicly available datasets were analyzed in this study. This data can be found here: https://www.chembridge.com/screening_libraries.

## Ethics Statement

The animal study was reviewed and approved by the animal laboratory of KIRAMS ethical approval (#2018-0062).

## Author Contributions

A-YK, JO, and J-SK conceived and designed the experiments and wrote the manuscript. A-YK, YY, and JL performed the experiments. K-YJ chemical synthesis. A-YK, JL, JO, and J-SK analyzed the data. J-YL performed *in silico* analysis. MK, JA, JO, and S-GH provided advice. All authors contributed to the article and approved the submitted version.

## Conflict of Interest

The authors declare that the research was conducted in the absence of any commercial or financial relationships that could be construed as a potential conflict of interest.
