# Supplementary material for: MKI-1, a Novel Small-Molecule Inhibitor of MASTL, Exerts Antitumor and Radiosensitizer Activities Through PP2A Activation in Breast Cancer
Source: Front Oncol. 2020 Sep 29;10:571601. doi: 10.3389/fonc.2020.571601 (PMC7550800; doi:10.3389/fonc.2020.571601)
Supplement: Supplementary file 1 [file Data_Sheet_1.doc]

**Supplementary Figure Legends**


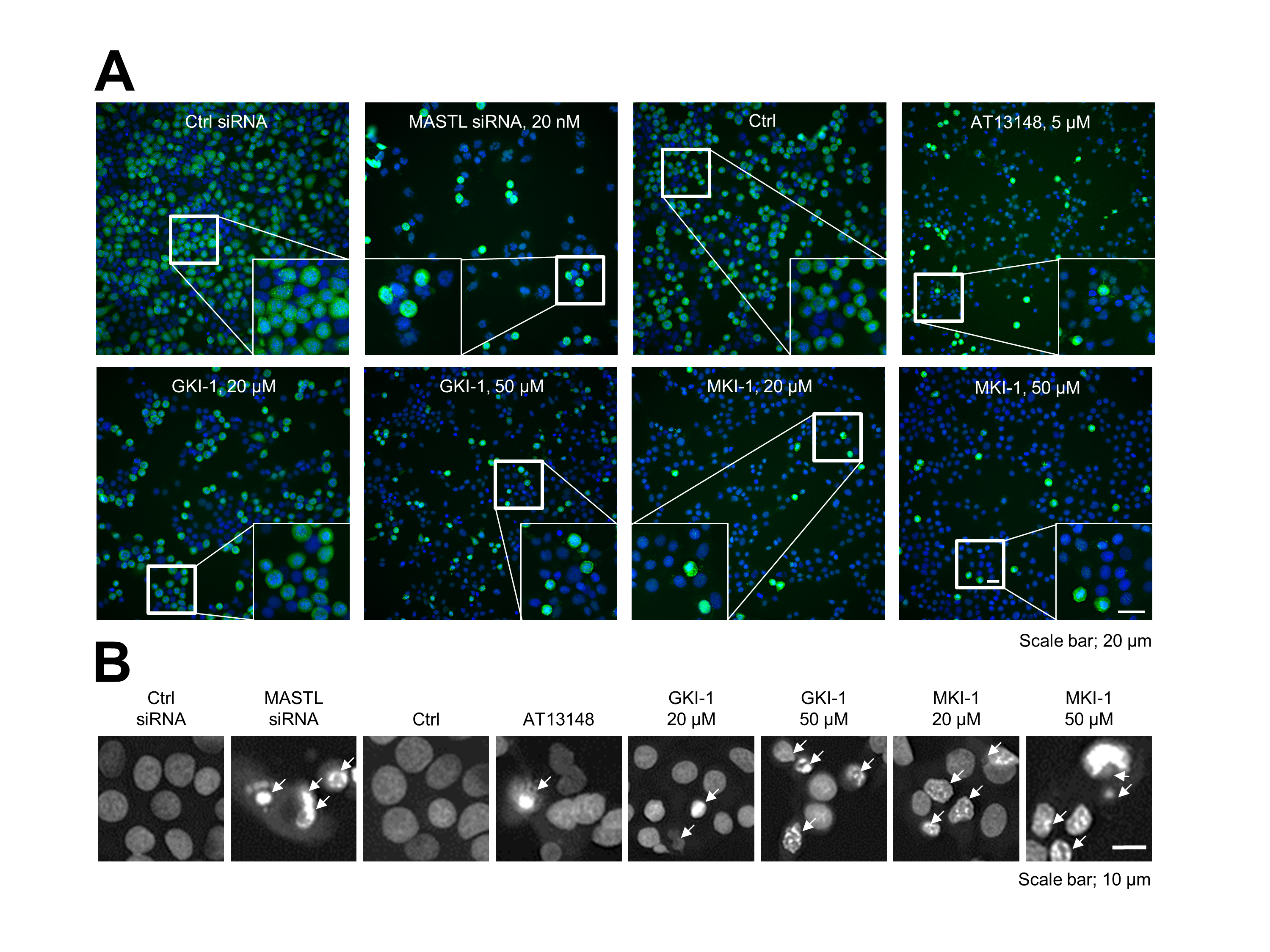


**Supplementary Figure 1.** Representative immunofluorescence images for p-ENSA (A) and aberrant nuclear cells (B).


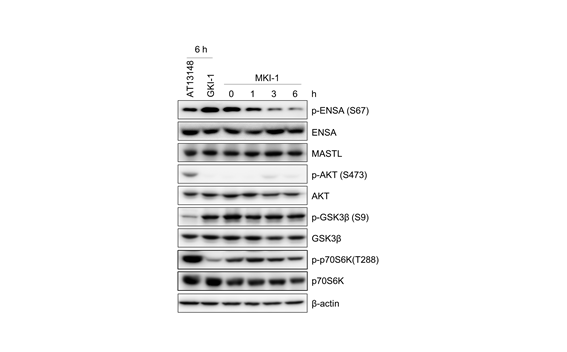


**Supplementary Figure 2.** MKI-1 inhibits phosphorylated ENSA in MCF7 cells. MCF7 cells were treated with 5 μM AT13148, 15 μM GKI-1, and MKI-1 for the indicated time points. The cell lysates were analyzed by immunoblotting for the indicated antibodies; β-actin was used as the loading control. The data represent typical results and are presented as the mean ± standard deviation of three independent experiments. *P < 0.01.
